# Lack of genetic susceptibility in takotsubo cardiomyopathy: a case-control study

**DOI:** 10.1186/s12881-018-0544-6

**Published:** 2018-03-07

**Authors:** Emma Mattsson, Peter Saliba-Gustafsson, Ewa Ehrenborg, Per Tornvall

**Affiliations:** 1 Department of Clinical Science and Education Södersjukhuset, Karolinska Institutet, Sjukhusbacken 10, 11883 Stockholm, Sweden; 2 Department of Medicine, Solna Center for Molecular Medicine (CMM), Karolinska University Hospital L8:03, Karolinska Institutet, Stockholm, Sweden

**Keywords:** Takotsubo cardiomyopathy, Polymorphisms, Single nucleotide, Receptors, Adrenergic, Bcl-associated athanogene 3

## Abstract

**Background:**

Takotsubo cardiomyopathy (TCM), also known as “broken heart syndrome”, is a type of heart failure characterized by transient ventricular dysfunction in the absence of obstructive coronary lesions. Although associated with increased levels of catecholamines, pathophysiological mechanisms are unknown. Relapses and family heritability indicate a genetic predisposition. Several small studies have investigated associations between three different loci; the β1-adrenic receptor (ADRB1), G-protein-coupled receptor kinase 5 (GRK5), Bcl-associated athanogene 3 (BAG3) and TCM but no consensus has been reached.

**Methods:**

Participants were recruited using the Swedish Coronary Angiography and Angioplasty Register (SCAAR). TCM patients without coronary artery disease (CAD)(*n* = 258) were identified and age- and sex-matched subjects with (*n* = 164) and without (*n* = 243) CAD were selected as controls. DNA was isolated from saliva and genotyped for candidate single nucleotide polymorphisms in the ADRB1, GRK5 and BAG3 genes. Allele frequencies and Odds Ratios (OR) with 95% Confidence Intervals (CI) for the investigated polymorphisms were compared, respectively calculated for TCM patients and controls.

**Results:**

There were no differences in allele frequencies between TCM patients and controls. OR (CI) for TCM patients having at least one minor allele using controls as reference were 1.07 (0.75–1.55) for ADRB1, 0.45 (0.11–1.85) for GRK5 and 1.27 (0.74–2.19) for BAG3.

**Conclusion:**

By genotyping a large takotsubo cohort, we demonstrate a lack of association between candidate SNPs in the ADRB1, GRK5 and BAG3 genes, earlier suggested to contribute to TCM. Our result indicates a need to expand the search for new genetic candidates contributing to TCM.

**Electronic supplementary material:**

The online version of this article (10.1186/s12881-018-0544-6) contains supplementary material, which is available to authorized users.

## Background

Takotsubo cardiomyopathy (TCM), or “broken heart syndrome”, is a form of acute and transient cardiac failure [1] typically characterized by left ventricular dysfunction in the absence of corresponding obstructive coronary lesions or ruptured plaques [2]. Frequent symptoms of TCM include dyspnoea, chest pain and syncope [3]. Additional clinical features include electrocardiographic changes, such as ST-segment elevation and prolongation of QT interval, and elevated troponin levels [4]. Due to similar clinical presentation, distinction between TCM and acute coronary syndromes (ACS) requires coronary angiography [5]. Among patients presenting with symptoms of ACS, approximately 2% are diagnosed with TCM [5, 6].

Although the pathophysiological mechanisms underlying TCM are unknown, the condition commonly affects women with approximately 10% of reported cases being males [5] and disease presentation is often preceded by a stressful incident [3]. Acutely elevated plasma levels of catecholamines in TCM patients [7] and that intravenous administration of synthetic catecholamines in patients with suspected coronary artery disease (CAD) trigger the syndrome [8] suggest that exaggerated sympathetic stimulation might contribute to TCM. A rat model demonstrating development of transient myocardial hypokinesia typical for TCM after intravenous administration of epinephrine supports this hypothesis [9].

Multiple cases of TCM in the same family [10] and recurrent cases [11] indicate a genetic component. Current literature has focused on the possible existence of associations between TCM and single nucleotide polymorphisms (SNPs) in genes associated with sympathetic stress. While some studies have presented associations between TCM and functional polymorphisms, leading to exchange of amino acids, in the β1-adrenergic receptor (ADRB1) [12] and the G-protein-coupled receptor kinase 5 (GRK5) [13], which regulate cardiac sympathetic stimulation, others have not been able to replicate these findings [14, 15]. Recently, genetic variation affecting the regulatory function of the anti-apoptotic protein Bcl-associated athanogene 3 (BAG3), thought to contribute to stress resistance of myocytes, has been proposed to contribute to TCM [16].

There is no consensus regarding the influence of polymorphisms in the ADRB1 and GRK5 genes on TCM development and, to the best of our knowledge, only one study supporting a possible association between BAG3 polymorphisms and TCM has been published. Existing studies of the influence of these polymorphisms on TCM have had small sample sizes and un-matched controls. The aim of this study was therefore to investigate the possible associations between previously identified SNPs in the ADRB1, GRK5 and BAG3 genes, and TCM in a large cohort with matched controls.

## Methods

### Study groups

Participants, including cases and controls, were recruited using the Swedish Coronary Angiography and Angioplasty Register (SCAAR) [17]. TCM patients registered in SCAAR were diagnosed using the Mayo Clinic diagnostic criteria [2]. Patients for the present study were enrolled from 2009 to 2013.

The case group consisted of TCM patients. Inclusion criteria were angiographic examination with subsequent definite TCM diagnosis. Exclusion criteria were death until the end of 2013, coronary stenosis and suspected TCM diagnosis. The aim was to, for every TCM patient, include two sex and age-matched controls. One control group consisted of patients with CAD (CAD controls). Inclusion criteria were ACS diagnosed with stenosis at coronary angiography followed by treatment with percutaneous coronary intervention. Exclusion criterion was death until 2013. The second control group consisted of patients without CAD (controls without CAD). Inclusion criterion was angiographic examination due to chest pain. Exclusion criteria were stenosis, history of myocardial infarction and death.

Initially, 1319 patients were requested to participate; 461 TCM patients, 403 controls with CAD and 455 controls without CAD. In order to increase the amount of controls without CAD, 119 additional requests were sent out to patients matching the inclusion criteria at a later occasion. Hence, the total amount of individuals requested to participate was 1438. Due to difference in mortality between the study groups, the participants became unevenly distributed. Ethical application for this study was approved by The Regional Ethical Review Board in Stockholm (https://www.epn.se/stockholm).

### Data acquisition

A request to participate, a questionnaire, a salivary test tube and an addressed envelope for return were sent by mail to all individuals invited to participate. The patients were asked to answer the questionnaire, donate a salivary sample and to sign a written informed consent form. Instructions were given to return the material using the attached envelope. The patients were informed about the voluntary participation. No reminders were sent out.

#### Materials for data acquisition

The participants were asked to respond to the following questions regarding their health profile at disease presentation; medication for hypertension, hyperlipidaemia and diabetes mellitus, smoking habit, experience of emotional/physical stress, family history of myocardial infarction or TCM. The participants were also asked to report any history of surgical procedures, comorbidity and medication at disease presentation (Additional file 1: Questionnaire). The salivary test tube used was Orangene-DNA® from DNAgenotek.

### Data registration and analysis

**Fig. 1:**
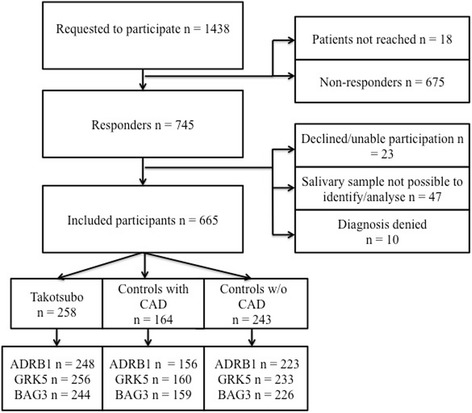
CONSORT diagram. Participants were included if accepting to participate and donated a salivary sample useful for DNA analysis. ADRB1 = β1-adrenergic receptor; BAG3 = Bcl-associated athanogene 3; GRK5 = G-protein-coupled receptor kinase 5

Participants were included if accepting participation and donating a salivary sample. The response rate, defined as acceptance to participate including the contribution of a salivary sample used for DNA isolation, was 46.2% in total. The response rates for the study groups were 56% for TCM patients, 40.7% for CAD controls and 42.3% for controls without CAD respectively. The drop-out was due to several factors presented in Fig. 1. The questionnaires and the salivary samples were de-identified and coded according to study group belonging.

#### Purification and genotyping of DNA from salivary samples

DNA was purified using prepIT-L2P kits (DNAgenotek) according to the manufacturer’s instructions. Briefly, purification was initiated by heat incubation at 50 °C for 2 h followed by adding of PT-L2P buffer, incubation in an ice-bath and centrifugation at room temperature for 20 min whereupon the supernatant was mixed with 95% ethanol. The DNA pellet was then centrifuged with 70% ethanol at room temperature for 5 min, dried, rehydrated with TE buffer (10 mM Tris-HCl, 1 mM EDTA, pH 8.0), incubated in water-bath at 50 °C for 3 h and finally incubated over-night in room temperature.

The samples were genotyped for the following polymorphisms: rs1801253 (Arg389Gly) for ADRB1, rs2230345 (Gln41Leu) for GRK5 and rs8946 (g.31131G > C, 3’-UTR) for BAG3. Genotyping was carried out on StepOnePlus Real-Time PCR System using TaqMan genotyping Master Mix (AppliedBiosystems) with rs1801253 (C__8898494_10), rs2230345 (C_189589070_10) and rs8946 (C__3174239_20) genotyping assays from Life Technologies. Failure to determine genotype occurred in 38 subjects for ADRB1 (10 TCM patients [4%], 8 controls with CAD [5%] and 20 controls without CAD [8%]), in 16 subjects for GRK5 (2 TCM patients [1%], 4 controls with CAD [2%] and 10 controls without CAD [4%]) and in 36 subjects for BAG3 (14 TCM patients [5%], 5 controls with CAD [3%], and 17 controls without CAD [7%]). Hardy-Weinberg equilibrium was calculated for the studied polymorphisms.

### Statistics

Descriptive statistics, including mean and standard deviation (SD) were used to describe the continuous variable age. Categorical variables were described with number (percentage). Due to the nature of the data (categorical variables) chi-square test and logistic regression were used to test for differences. Odds-ratio (OR) and confidential interval (CI) were calculated for the probability of TCM patients and CAD controls having at least one rare allele or being homozygous for the rare allele, respectively. Controls without CAD were used as the reference group. The tests were two-sided and a *p*-value < 0.05 was considered significant. The results were analysed using SPSS® statistics version 23.

## Results

### Patient characteristics

Mean age and female proportion for the included study groups were 68 years (SD 9.6) and 90.7% for TCM patients, 67 years (SD 8.9) and 85.4% for CAD controls and 67 years (8.4) and 89.7% for controls without CAD, respectively.

Characteristics of TCM patients, sex and age-matched controls with or without CAD are presented in Table 1. TCM patients were less likely to have diabetes mellitus and smoke but more likely to medicate with cortisone than CAD controls. TCM patients were less likely to have hyperlipidaemia but more likely to smoke than controls without CAD. TCM patients were more likely to have experienced stress during disease manifestation than controls with or without CAD.

**Table 1:** Frequencies of patient characteristics and Pearsons or Fishers chi-square test of association between each characteristic and takotsubo cardiomyopathy in 258 patients with Takotsubo cardiomyopathy and sex and age-matched controls with and without coronary artery disease

Variable	TCM		Controls with CAD		Controls w/o CAD		*P*-Value	
	(*n* = 258)		(*n* = 164)		(*n* = 243)		TCM	TCM
	Number (%)		Number (%)		Number (%)		vs Controls	vs Controls
		Response rate (%)		Response rate (%)		Response rate (%)	with CAD	w/o CAD
Gender								
Men	24 (9.3%)	100	24 (14.6%)	100	25 (10.3%)	100		
Women	234 (90.7%)	100	140 (85.4%)	100	218 (89.7%)	100		
Characteristics								
Smoker	39 (15.3%)	98.8	51 (31.3%)	99.4	13 (5.4%)	98.4	*p* < 0.001	*p* < 0.001
Erlier smoker	99 (43.6%)	88	49 (34%)	87.8	102 (46.8%)	84.5	n.s.	n.s.
Heritability TCM	12 (5%)	93.4	39 (27.5%)	86.6	30 (15.4%)	80.2	*p* < 0.001	*p* < 0.001
Heritability CHD	66 (26.6%)	96.1	60 (38%)	96.3	81 (35.2%)	94.7	*p* < 0.05	*p* < 0.05
Stress	185 (72.5%)	98.8	70 (43.2%)	98.8	117 (49.6%)	97.1	*p* < 0.001	*p* < 0.001
Emotional stress	176 (70.1%)	97.3	60 (37%)	98.8	100 (43.1%)	95.5	*p* < 0.001	*p* < 0.001
Physical stress	15 (6%)	97.3	3 (1.9%)	98.8	19 (8.2%)	95.5	*p* < 0.05	n.s.
Nordic origin	245 (92.2%)	95	152 (94.7%)	92.7	226 (95.6%)	93	n.s.	n.s.
Co-morbidity								
Hypertension	98 (39.5%)	96.1	63 (40.1%)	95.7	110 (47.2%)	95.9	n.s	n.s
Hyperlidemia	45 (18%)	96.9	39 (24.2%)	98.2	64 (27.6%)	95.5	n.s	*p* < 0.05
Diabetes mellitus	17 (6.7%)	98.4	24 (14.6%)	100	14 (5.8%)	98.8	*p* < 0.01	n.s
Asthma	10 (3.9%)	98.8	7 (4.3%)	99.4	15 (6.2%)	99.2	n.s	n.s
Chronic obstructive pulmonary disease	10 (3.9%)	98.8	3 (1.8%)	99.4	4 (1.7%)	99.2	n.s	n.s
Reumatism	9 (3.6%)	96.9	4 (2.5%)	98.2	11 (4.6%)	97.9	n.s	n.s
Hypothyroidism	24 (9.4%)	98.8	11 (6.8%)	98.8	16 (6.6%)	99.2	n.s	n.s
Medication								
Medication during disease presentation	170 (67.2%)	98	96 (58.9%)	99.4	169 (72.5%)	95.9	n.s	n.s
Beta blockers	34 (13.5%)	97.7	22 (13.7%)	98.2	45 (19.5%)	95.1	n.s	n.s
ACE inhibitors	21 (8.3%)	97.7	9 (5.6%)	98.2	17 (7.4%)	95.1	n.s	n.s
ARBs	24 (9.5%)	97.7	8 (5%)	98.2	19 (8.2%)	95.1	n.s	n.s
Diuretics	16 (6.3%)	97.7	10 (6.2%)	98.2	21 (9.1%)	95.1	n.s	n.s
Antikoagulantia	29 (11.5%)	97.7	17 (10.6%)	98.2	31 (13.4%)	95.1	n.s	n.s
Psyciatric drugs	28 (11.1%)	97.7	8 (5%)	98.2	11 (4.8%)	95.1	*p* < 0.05	*p* < 0.05
Cortisone	11 (4.4%)	97.7	0 (0%)	98.2	8 (3.5%)	95.1	*p* < 0.01	n.s

Abbreviations: *TCM* Takotsubo cardiomyopathy; *CAD* coronary artery disease; *n.s* non-significant; *n* = number of participants. Frequencies were calculated from available responses

### Genotype distribution of the studied polymorphisms

Genotype distributions of the studied polymorphisms are presented in Table 2. All genotype distributions adhered to Hardy-Weinberg equilibrium (Additional file 2: Table S1). The genotype distribution did not differ between the three study groups.

**Table 2:** Genotype distributions of ADRB1, GRK5 and BAG3 gene

Genotype ADRB1	TCM	Controls with CAD	Controls w/o CAD
	(*n* = 248)	(*n* = 156)	(*n* = 223)
CC	138 (55.6%)	90 (57.7%)	128 (57.4%)
CG	93 (37.5%)	53 (34%)	79 (35.4%)
GG	17 (6.9%)	13 (8.3%)	16 (7.2%)
Genotype GRK5	TCM	Controls with CAD	Controls w/o CAD
	(*n* = 256)	(*n* = 160)	(*n* = 233)
AA	253 (98.8%)	155 (96.9%)	227 (97.4%)
AT	3 (1.2%)	5 (3.1%)	6 (2.6%)
TT	0 (0%)	0 (0%)	0 (0%)
Genotype BAG3	TCM	Controls with CAD	Controls w/o CAD
	(*n* = 244)	(*n* = 159)	(*n* = 226)
CC	28 (11.5%)	14 (8.8%)	14 (8.8%)
GC	118 (48.4%)	78 (49.1%)	99 (43.8%)
GG	98 (40.2%)	67 (42.1%)	95 (42%)

Abbreviations: *TCM* Takotsubo cardiomyopathy; *CAD* coronary artery disease; *ADRB1* = β1-adrenergic receptor; *GRK5* G-protein-coupled receptor kinase 5; *BAG3* Bcl-associated athanogene 3

### Allele frequencies in the different study groups

The allele frequencies of the studied polymorphisms did not differ between the three study groups. For the minor G-allele variant of ADRB1 Arg389Gly (rs1801253), the allele frequencies were 0.26 for TCM, 0.25 for CAD controls and 0.25 for controls without CAD. For the minor T-allele variant of GRK5 Gln41Leu (rs2230345), the allele frequencies were 0.01 for TCM, 0.02 for CAD controls and 0.01 for controls without CAD. For the minor C-allele of BAG3 g.31131G > C, 3’-UTR (rs8946), the allele frequencies were 0.36 for TCM, 0.33 for CAD controls and 0.36 for controls without CAD. The frequencies of all three polymorphisms are consistent with reference populations in the 1000 Genomes database. Using logistic regression analysis, we found that none of the studied polymorphisms were associated with TCM (Table 3). ORs for TCM patients and controls with CAD having at least one minor allele were 1.07 respectively 0.99 for ADRB1, 1.27 respectively 1.71 for BAG3 and 0.45 respectively 1.22 for GRK5. ORs for TCM patients and controls with CAD being homozygous for the minor allele were 0.95 respectively 1.18 for ADRB1 and 0.79 respectively 0.59 for BAG3. Controls without CAD were used as reference group.

**Table 3:** OR and CI for TCM patients and controls with CAD being heterozygote and homozygote for minor allele in the ADRB1 (G), GRK5 (T) and BAG3 (C) gene

Polymorphism	TCM OR (CI)	CAD Controls OR (CI)
Heterozygote for minor allele		
β1AR	1.07 (0.75–1.55)	0.99 (0.65–1.50)
BAG3	1.27 (0.74–2.19)	1.71 (0.88–3.32)
GRK5	0.45 (0.11–1.85)	1.22 (0.37–4.07)
Homozygote for minor allele		
β1AR	0.95 (0.47–1.93)	1.18 (0.55–2.52)
BAG3	0.79 (0.46–1.35)	0.59 (0.3–1.14)

Abbreviations: *TCM* Takotsubo cardiomyopathy; *CAD* coronary artery disease; *ADRB1* = β1-adrenergic receptor; *GRK5* G-protein-coupled receptor kinase 5; *BAG3* Bcl-associated athanogene 3

## Discussion

By using a national register, covering all patients investigated by coronary angiography in Sweden with information about TCM (SCAAR) for participant enrolment, we have performed the so far largest genetic study of TCM patients with age- and sex-matched controls with and without CAD. We investigated the prevalence of polymorphisms earlier reported to be associated with TCM. When analyzing DNA from 258 TCM patients, we found no associations between candidate SNPs in the ADRB1, GRK5 and BAG3 gene and TCM using control subjects without CAD as reference.

Since experience of stress is frequently reported to trigger TCM, exaggerated sympathetic cardiac stimulation has been discussed as a possible physiological mechanism behind TCM. In a genetic study including 61 TCM patients, Vriz et al. presented an association between the same ADRB1 polymorphism as investigated in the current study and TCM [12], suggesting that genetic variation influencing cardiac sympathetic stimulation might contribute to TCM. This hypothesis is supported by studies indicating that the Arg389 variant of the ADRB1 has a higher sensitivity to catecholamine stimulation compared with the Gly389 variant [18]. In our study group, the Arg389 variant was the most common allele, validating a finding recently observed by an Australian study [14]. In line with the same Australian study, we found no association between the ADRB1 polymorphism (rs1801253) and TCM. Due to differences in SNP distribution between ethnic groups [19, 20], comparison with controls of a different ethnic group than the TCM patients could have affected the results in the study by Figtree et al. [14]. Both TCM patients and controls included in the study by Vriz et al. were Caucasians while Figtree et al. used Australian TCM patients and American controls with ancestry from Europe. Although intending to minimize the impact of ethnical diversity by including only people living in Sweden in the current study, we could not replicate the findings made by Vriz et al. One possible explanation could be failure in controlling for a diverse ethnical distribution of genotypes in the current study. However, this risk is considered small since the allele frequencies observed in our study were similar to those found in TCM patients in the Australian study [14] and that failure in controlling for a diverse ethnical distribution of genotypes would have required a large number of participants with different ethnicities. In addition, allele distributions observed in our study are in agreement with those of a previous study of a Scandinavian population [21]. Hence, we believe the most likely explanation to the discrepancy between our results and the findings made by Vriz et al. is the difference in sample size.

Another way of influencing the cardiac stress response to catecholamines is GRK5 mediated desensitisation of β-adrenergic receptors. The Gln41Leu GRK5 polymorphism has been shown to affect βAR desensitisation during exposure to catecholamine stimulation [22] and has therefore been suggested to contribute to TCM [13]. Although genetic variation in GRK5 is a reasonable pathophysiological hypothesis, we found no association between this polymorphism and TCM. However, since the minor allele frequency is very low, finding of an association requires a larger sample size unless the impact of the polymorphism is very large.

Recently, the anti-apoptotic BAG3, shown to increase susceptibility to damage during catecholamine stress, has been presented as another genetic candidate [16]. In 2015 d’Avenia et al. [16] analyzed 6 BAG3 SNPs in 70 TCM patients and 81 healthy blood donors with no signs of cardiovascular disease. The most frequent SNP (rs8946), coding for a regulating microRNA [16], was found to be more common among TCM patients than controls with an absolute difference in allele frequency of more than 10%. The same SNP was proposed to potentially impair a signaling pathway leading to up-regulation of BAG3 in cardiomyocytes in response to epinephrine. In the current study the BAG3 SNP was genotyped and we were unable to validate the finding of an association between this SNP (rs8946) and TCM. Despite the larger sample size and greater power to detect an existing association between the polymorphism and TCM, than in the study of d’Avenia et al. [16], we were unable to corroborate the existence of such an association, which may suggest a spurious finding in their study.

Clinical characteristics associated with TCM in the current study were medication for psychiatric disease and experience of stress at disease manifestation. These findings validate other studies proposing psychiatric [23, 24] and stress [4] comorbidity in TCM. To our knowledge, no other studies have compared the prevalence of preceding stress among TCM patients with the prevalence in controls. The only cardiovascular risk factor observed in a higher degree among TCM patients than controls without CAD was smoking. Unexpectedly, controls with and without CAD were more likely to report heritability of TCM than TCM patients. This is probably due to misunderstanding of the question. In contrast to TCM patients, controls may not be familiar with the diagnosis “broken heart” and might have assumed we were referring to CAD.

Limitations of this study included an unknown representativity of the study groups for the general population. Since patients were recruited by mail there is a possibility of selection bias. Another limitation is that the controls without CAD might not have been healthy, the only examinations performed served to exclude CAD and previous myocardial infarction. A potential heterogeneity of this control group might have influenced the results. The use of self-reported questionnaires for data acquisition entails a risk for self-report bias, which might decrease the internal validity of the current study. Furthermore, the call rates of the studied SNPs were low, possibly due to the origin of DNA. Although DNA purified from saliva has been reported to be of similar quality as blood DNA it cannot be excluded that some of the samples were of poor quality. Although we have performed the largest genetic study of TCM patients so far, the number of subjects included is still low. The power of the present study allowed us to detect an association between the two common polymorphisms and TCM only if the minor allele frequencies differ more than ten absolute percent when the TCM group is compared to any of the control groups. In order to provide strong evidence for genetic associations, a larger sample size is required. Future genome-wide association studies should be done to search for unknown genetic variants associated with TCM. Preferably, all included participants should belong to the same ethnic group. This arrangement would provide the best possible conditions for discovering genetic predisposition to TCM.

## Conclusion

By performing the so far largest genetic study of TCM, we were unable to demonstrate an association between the proposed candidate SNPs in the ADRB1, GRK5 and BAG3 genes, and TCM. This strongly indicates a need for expanding the search in order to clarify the potential genetic predisposition to TCM, preferably using a genome-wide approach. Discovering genetic variation explaining disease susceptibility might entail both the understanding of intracellular signalling dysfunction among TCM patients and give future treatment suggestions.

## Additional files

Additional file 1: Questionnaire. Questionnaire, translated from Swedish to English, sent to patients and controls to recover medical history. (DOCX 12 kb)

Additional file 2: **Table.** Allele frequencies. Chi square test of observed and expected allele frequencies. (DOCX 91 kb)
