# Supplementary material for: Lack of genetic susceptibility in takotsubo cardiomyopathy: a case-control study
Source: BMC Med Genet. 2018 Mar 7;19:39. doi: 10.1186/s12881-018-0544-6 (PMC5842616; doi:10.1186/s12881-018-0544-6)
Supplement: Supplementary file 1 — Questionnaire. Questionnaire, translated from Swedish to English, sent to patients and controls to recover medical history. (DOCX 12 kb) [file 12881_2018_544_MOESM1_ESM.docx]

**Name: Date:**

**Personal identification number:**

Did you smoke at admission? Yes No

Were you a previous smoker at admission? Yes No

Were you on anti-hypertensive treatment at admission? Yes No

Were you on lipid-lowering treatment at admission? Yes No

Were you on anti-diabetic treatment at admission? Yes No

Do you have a first degree relative with “broken heart”? Yes No

Do you have a first degree relative with myocardial infarction

before 65 years of age? Yes No

Did you experience mental or physical stress at admission? Yes No

If yes, can you describe the stress:

Have you had any major surgeries previously? Yes No

If yes, can you describe the operation and year:

Did you have a chronic disease at admission? Yes No

If yes, can you describe the disease:

(for example rheumatoid arthritis, chronic obstructive pulmonary disease or cancer)

Were you on any regular medication at admission? Yes No

If yes, can you write down your medication:
