# Supplementary material for: Lack of genetic susceptibility in takotsubo cardiomyopathy: a case-control study
Source: BMC Med Genet. 2018 Mar 7;19:39. doi: 10.1186/s12881-018-0544-6 (PMC5842616; doi:10.1186/s12881-018-0544-6)
Supplement: Supplementary file 2 — Table. Allele frequencies. Chi square test of observed and expected allele frequencies. (DOCX 91 kb) [file 12881_2018_544_MOESM2_ESM.docx]

ADRB1 = β1-adrenergic receptor, GRK5 = G-protein-coupled receptor kinase
5, BAG3 = Bcl-associated athanogene 3.
